# Is *Toxoplasma gondii* Infection Associated with Sexual Promiscuity? A Cross-Sectional Study

**DOI:** 10.3390/pathogens10111393

**Published:** 2021-10-28

**Authors:** Cosme Alvarado-Esquivel, Sergio Estrada-Martínez, Agar Ramos-Nevárez, Alma Rosa Pérez-Álamos, Isabel Beristain-Garcia, Ángel Osvaldo Alvarado-Félix, Sandra Margarita Cerrillo-Soto, Gustavo Alexis Alvarado-Félix, Carlos Alberto Guido-Arreola, Leandro Sáenz-Soto, Antonio Sifuentes-Álvarez

**Affiliations:** 1 Biomedical Research Laboratory, Faculty of Medicine and Nutrition, Juárez University of Durango State, Durango 34000, Mexico; 1158056@alumnos.ujed.mx (Á.O.A.-F.); gustavo_alvarado_19@anglodurango.edu.mx (G.A.A.-F.); dr_sifuentes@ujed.mx (A.S.-Á.); 2 Institute for Scientific Research “Dr. Roberto Rivera-Damm”, Juárez University of Durango State, Durango 34000, Mexico; sergio.estrada@ujed.mx (S.E.-M.); alma.perez@ujed.mx (A.R.P.-Á.); 3 Clínica de Medicina Familiar, Instituto de Seguridad y Servicios Sociales de los Trabajadores del Estado, Durango 34079, Mexico; agar_ramos@hotmail.com (A.R.-N.); agar.ramos@issste.gob.mx (S.M.C.-S.); cguido@issste.gob.mx (C.A.G.-A.); quim_saenz@hotmail.com (L.S.-S.); 4 Facultad de Enfermería y Obstetricia, Juárez University of Durango State, Durango 34000, Mexico; isabel.beristain@ujed.mx

**Keywords:** *Toxoplasma gondii*, sexual promiscuity, cross-sectional study, seroprevalence, behavior, epidemiology

## Abstract

We determined the association between *T. gondii* seropositivity and a history of sexual promiscuity. The study included 3933 people (mean age: 41.81 ± 14.31 years) who attended public health facilities. Face-to-face interviews were used to collect data. Enzyme immunoassays were used to determine anti-*T. gondii* IgG and IgM antibodies. Anti-*T. gondii* IgG antibodies were found in 57 (18.1%) of 315 individuals with sexual promiscuity and in 374 (10.3%) of 3618 individuals without this practice (OR: 1.91; 95% CI: 1.41–2.60; *p* < 0.0001). High (>150 IU/mL) levels of anti-*T. gondii* IgG antibodies were found in 29 (9.2%) of the 315 participants with sexual promiscuity and in 167 (4.6%) of the 3618 participants without this history (OR: 2.09; 95% CI: 1.38–3.16; *p* = 0.0003). The association of sexual promiscuity with *T. gondii* seropositivity and serointensity was observed in men but not in women. Sexual promiscuity was associated with *T. gondii* seropositivity in all age groups studied (≤30 years, 31–50 years, and >50 years) and with *T. gondii* serointensity in two age groups (≤30 years, and >50 years). No difference in the frequencies of anti-*T. gondii* IgM antibodies among the groups was found. Our findings indicate that *T. gondii* seropositivity and serointensity are associated with sexual promiscuity.

## 1. Introduction

The parasite *Toxoplasma gondii* (*T. gondii*) is an intracellular protozoan that can infect, survive, and replicate in nearly all mammalian cells [1]. Nearly one-third of humanity has been infected with *T. gondii* [2]. Toxoplasmosis, a disease caused by infection with *T. gondii*, is a zoonosis with medical and veterinary importance [3]. However, this disease is still a neglected parasitic infection [1]. As the most common feline definitive host, cats play a vital role in the transmission of *T. gondii* [4]. The main route of infection with *T. gondii* is consumption of raw or undercooked meat containing tissue cysts [2]. Infection with *T. gondii* can also occur by ingestion of water or food contaminated with oocysts from the feces of infected cats [5]. Most human infections with *T. gondii* are mild or asymptomatic [1]. However, individuals infected with *T. gondii* may develop ocular disease or cervical lymphadenopathy [5]. Reactivation of a latent infection in immune deficiency conditions can cause fatal toxoplasmic encephalitis, and primary infection with *T. gondii* in pregnant women may cause health-threatening sequelae for the fetus or death in uterus [6]. Recent studies suggest that asymptomatic infections with *T. gondii* may have effects on behavior [7] and might be responsible for a vast array of neuropsychiatric symptoms [8]. *T. gondii* can persist lifelong in the central nervous system within neurons, modifying their function and structure and leading to specific behavioral changes of the host [9]. Infections with *T. gondii* have been associated with schizophrenia [10,11], suicide behavior [12,13,14], mixed anxiety and depressive disorder [15], traffic accidents [16], and aggression [17]. In contrast, some studies have shown a lack of association between *T. gondii* seropositivity and depression in pregnant women [18], completed suicide [19], and bipolar disorder [20]. Reports thus indicate that further research to elucidate the link between *T. gondii* infection and clinical and behavioral changes are largely needed. It is unclear whether *T. gondii* infection might be linked to sexual behavior. We hypothesized that sexual promiscuity is associated with *T. gondii* infection. On the one hand, a group of researchers have hypothesized that toxoplasmosis can be a sexually transmitted infection [21]. If *T. gondii* can be transmitted by sexual intercourse, persons with sexual promiscuity might have a higher risk for *T. gondii* infection than persons without this practice. High seroprevalence of *T. gondii* infection has been reported in population groups with large number of sexual partners or having a sexually transmitted infection including female sex workers [22] and people with human immunodeficiency virus infection [23]. On the other hand, high seroprevalence of infection with *T. gondii* is found in psychiatric patients [11,24] and sexual promiscuity is a common behavior in patients with psychiatric illnesses. An increase in sexual activity and promiscuity have been found in patients with bipolar depression [25], depressed children [26], patients with borderline personality disorder [27], and psychiatric patients with a history of suicide attempts [28]. In a study of 137 psychiatric inpatients in Mexico, an association between *T. gondii* seropositivity and sexual promiscuity was found [24]. However, in such a study, the analysis was performed in a quite limited number of psychiatric inpatients (not in their controls), in a single hospital, and the association was obtained without any matching or stratification by age and gender. Therefore, in the present study including a large sample of participants, in several public health facilities, and with a stratification by age and gender, we aimed to determine the association between *T. gondii* seropositivity and sexual promiscuity.

## 2. Results

Of the 3933 people surveyed, 315 (8.0%) had a history of sexual promiscuity and 3618 (92.0%) did not have this history. Anti-*T. gondii* IgG antibodies were found in 57 (18.1%) of the 315 individuals with a history of sexual promiscuity and in 374 (10.3%) of the 3618 individuals without this history (OR: 1.91; 95% CI: 1.41–2.60; *p* < 0.0001). Logistic regression analysis showed that the association between *T. gondii* infection and sexual promiscuity remained significant when an adjustment by age, gender, and residence area (urban vs. rural) was performed (OR: 1.54; 95% CI: 1.11–2.14; *p* = 0.009). Table 1 shows a stratification by age and gender and seroprevalence of *T. gondii* infection in individuals with and without a history of sexual promiscuity.

Men with a history of sexual promiscuity had a significantly higher (30/94: 31.9%) seroprevalence of *T. gondii* infection than men without this history (104/748: 13.9%) (OR: 2.90; 95% CI: 1.79–4.69; *p* < 0.0001). Participants of the age groups ≤30 years, 31–50 years, and >50 years with a history of sexual promiscuity had a significantly higher seroprevalence of *T. gondii* infection than those of the same age group without sexual promiscuity (*p* = 0.004; *p* = 0.02; and *p* = 0.01, respectively). High (>150 IU/mL) levels of anti-*T. gondii* IgG antibodies were found in 29 (9.2%) of the 315 participants with a history of sexual promiscuity and in 167 (4.6%) of the 3618 participants without this history (OR: 2.09; 95% CI: 1.38–3.16; *p* = 0.0003). Table 2 shows a stratification by sex and age groups and the association between high (>150 IU/mL) anti-*T. gondii* IgG antibody levels and sexual promiscuity.

The rate of high (>150 IU/mL) levels of anti-*T. gondii* IgG antibodies was higher in men with sexual promiscuity than in men without this practice (OR: 3.36; 95% CI: 1.77–6.35; *p* < 0.0001). Participants of the age groups ≤30 years and >50 years with a history of sexual promiscuity had a significantly higher rate of high (>150 IU/mL) levels of anti-*T. gondii* IgG antibodies than those of the same age groups without sexual promiscuity (*p* = 0.02; and *p* = 0.04, respectively).

Anti-*T. gondii* IgM antibodies were found in 23 (40.4%) of the 57 participants with anti-*T. gondii* IgG antibodies and a history of sexual promiscuity and in 118 (31.6%) of the 374 individuals with anti-*T. gondii* antibodies and without sexual promiscuity (OR: 1.46; 95% CI: 0.82–2.60; *p* = 0.18).

## 3. Discussion

There is scarce information about the link between *T. gondii* infection and sexual behavior. Therefore, in this survey, we determined the association between *T. gondii* exposure and sexual promiscuity in a large sample of people attending public health facilities in Durango, Mexico. We found that individuals with sexual promiscuity had a significantly higher seroprevalence of *T. gondii* infection than individuals without this practice. This higher seroprevalence of *T. gondii* infection was observed in all age groups studied. In addition, the rate of high levels of anti-*T. gondii* IgG antibodies was significantly higher in participants with sexual promiscuity than in those without this practice. This higher rate of high anti-*T. gondii* IgG antibodies was observed in two of the three age groups studied. Therefore, our findings suggest that *T. gondii* seroprevalence and serointensity is associated with sexual promiscuity. Our results are in line with the one reported in a seroepidemiology study of 137 psychiatric inpatients in Durango City, where an association between *T. gondii* seropositivity and sexual promiscuity was found [24]. However, in such a study, no stratification by age and sex was performed, and no association between sexual promiscuity and *T. gondii* serointensity or IgM seropositivity was assessed. In the present study, we performed a stratification by age groups in order to determine whether the observed association was uniform regardless of the age of participants or whether it occurred only in a certain age group. Seropositivity to *T. gondii* was associated with sexual promiscuity in all age groups, whereas *T. gondii* serointensity was associated with sexual promiscuity in people aged ≤30 years and >50 years but not in individuals aged 31–50 years. It is unclear why the *T. gondii* serointensity association was not uniformly observed in all age groups. It is possible that unknown factors linked to *T. gondii* infection and/or sexual promiscuity present in individuals aged 31–50 years differed from those present in younger and older individuals. Anti-*T. gondii* IgG antibodies peak at 3 months after infection and then remain at a plateau level for six months and after one year start to decrease [29]. A study of mice showed that increased IgG levels correlated with proliferation of tachyzoites in the brain during the chronic stage of infection, suggesting that high anti-*T. gondii* IgG antibody levels may indicate an occurrence of cerebral tachyzoite growth in immunocompetent individuals chronically infected with *T. gondii* [30]. Thus, in the present study, a considerable number of participants with high anti-*T. gondii* IgG antibody levels and sexual promiscuity might have had a recent infection or a reactivation of the infection. Anti-*T. gondii* IgM antibodies usually appear after one week of infection and disappear after 9 months but can remain for two years or more in some individuals [29]. Thus, the lack of association between *T. gondii* IgM seropositivity and sexual promiscuity found in the current study suggests that the association between *T. gondii* infection and sexual promiscuity occurred in individuals with chronic infections. A recent review on advances in *T. gondii* behavioral biology showed that infection with *T. gondii* alters host hormones and neurotransmission within the host brain, and that *T. gondii* effects could be mediated through neuroinflammation [31]. In another review, researchers showed that several factors interact and combine to alter the likelihood and outcome of clinical and neuropsychiatric disease including host genetics, parasite genotype, seroprevalence, and coinfecting neurotropic agents [32]. Intriguingly, in the present study, stratification by gender showed that the association between *T gondii* seropositivity and serointensity was present in men but not in women. It is not clear why this association was found only in men, but it raises the question whether testosterone might be playing any role in the association. Reports indicate that *T. gondii* increases the synthesis of testosterone as shown in a recent review [31], and this effect might lead to sexual promiscuity in *T. gondii*-infected individuals. In a recent article, researchers argued that the presence of tissue cysts in the host brain is merely incidental to the behavioral change and proposed a neuroendocrine loop to explain the role of gonadal steroids in the parasitized host in mediating the behavioral manipulation [33]. Some researchers have suggested that a higher level of testosterone could be responsible for at least some of the toxoplasmosis-associated shifts in human and animal behavior [34]. In a study on the association of latent toxoplasmosis and levels of serum testosterone in humans, researchers found a higher testosterone concentration in *T. gondii* seropositive individuals than in *T. gondii* seronegative ones [35]. In a study of students, researchers found that *T. gondii*-infected men had a higher concentration of testosterone and *T. gondii*-infected women had a lower concentration of testosterone than *T. gondii*-free controls [36]. In addition to testosterone, a possible mechanism by which *T. gondii* may affect human behavior is its effect on dopamine [37]. *T. gondii* contains two genes encoding tyrosine hydroxylase that catabolize phenylalanine to tyrosine and tyrosine to L-DOPA, and L-DOPA is the precursor to dopamine [38]. However, a study showed that infection with *T. gondii* did not affect host dopamine levels in vitro or in vivo [39]. Variation in the human dopamine D4 receptor gene has been associated with both sexual promiscuity and infidelity [40]. On the other hand, experiments in male rats have shown that *T. gondii* reprograms host brains to change fear to sexual attraction [41,42]. The results of the above-mentioned studies in humans and animals suggest that there is a link between *T. gondii* infection and sexual behavior. The results of our study may mean that infection with *T. gondii* could lead to sexual promiscuity, especially in men, or that people with sexual promiscuity could have risk factors that favor infection with *T. gondii*. Sexual promiscuity has been linked to mental health problems in men [43], and infection with *T. gondii* has been linked to mental disorders [10,11,12,13,14,15]. Therefore, sexual promiscuity might be a manifestation of mental disorders in some individuals exposed to *T. gondii*. In fact, in a previous report, *T. gondii* seropositivity was associated with sexual promiscuity in psychiatric inpatients [24]. On the other hand, whether *T. gondii* could be transmitted by sexual contact in people with sexual promiscuity is unclear. Sexual transmission of *T. gondii* is a matter of controversy. A study showed that transmission of *T. gondii* through the sexual route did not have epidemiological significance for men and animals [44]. In another study, a poor concordance of serological and molecular markers of *T. gondii* infection among heterosexual couples was found [45]. In contrast, a study showed that female sex workers had a higher seroprevalence of *T. gondii* infection than their controls, and injuries during sex work were associated with *T. gondii* seropositivity [22]. In a recent study of couples, the prevalence of toxoplasmosis in women with infected male partners was higher than in women with uninfected male partners, suggesting a sexual transmission of *T. gondii* from men to women [46]. The present study has limitations: (1) we performed a logistic regression analysis with adjustment of only few variables, and additional research with a more extensive analysis of variables should be conducted; and (2) we did not perform molecular analyses. It is of interest to determine the *T. gondii* genotypes in people in Mexico. It raises the question whether any *T. gondii* genotype might be linked to behavioral changes including sexual promiscuity. Further research to determine the association between *T. gondii* infection and sexual promiscuity using a case-control study design is needed. In addition, studies to determine the seroepidemiology of *T. gondii* infection in people with sexual promiscuity including analysis of sociodemographic, housing, clinical, and behavioral characteristics of participants should be conducted.

## 4. Materials and Methods

### 4.1. Study Design and Study Population

This cross-sectional study was conducted in Durango State, Mexico from June 2014 to May 2018. Individuals aged ≥15 years, of any gender, who attended 7 public health facilities in Durango City and rural Durango were invited to participate in the study. In total, 3933 people with a written informed consent were included in the survey. Of them, 842 were men and 3091 were women. The mean age of participants was 41.81 ± 14.31 (range 15–93) years. Face-to-face interviews were used to collect survey data. General data and a history of sexual promiscuity from participants were obtained.

### 4.2. Serology Tests

A blood sample was obtained from each participant. Sera were obtained from blood samples and stored at −20 °C until testing. Sera were tested for the presence of anti-*T. gondii* IgG antibodies using a commercially available enzyme immunoassay “*Toxoplasma gondii* IgG” kit (Diagnostic Automation/Cortez Diagnostics, Inc., Woodland Hills, CA, USA). This test was run in qualitative and quantitative formats. The manufacturer’s suggested positive cut-off of 8 IU/mL was used. According to the manufacturer’s insert, this IgG test has a sensitivity of 95.3%, a specificity of 100%, and an intra-run precision (C.V.) between 2.0% and 23.3%. High anti-*T. gondii* IgG antibody levels may indicate an acute or recent infection, or in chronic infections, reactivation or reinfection. Measuring the level of IgG can provide information about an on-going or resumed replication of the pathogen [47]. Sera that were positive for anti-*T. gondii* IgG antibodies were tested for detection of anti-*T. gondii* IgM antibodies by a commercially available enzyme immunoassay “*Toxoplasma gondii* IgM” kit (Diagnostic Automation/Cortez Diagnostics, Inc.). According to the manufacturer’s insert, this IgM test has a sensitivity of 100%, a specificity of 100%, an intra-run precision (C.V.) between 1.5% and 6.0%, and an inter-run precision (C.V.) between 8.0% and 12.3%. The good sensitivity, specificity, and precision of the IgG and IgM tests indicate that these tools are reliable for the diagnosis of *T. gondii* infection. All assays were performed according to the manufacturer’s instructions.

### 4.3. Statistical Analysis

Statistical analyses were performed using the software SPSS version 15 and EPIDAT 3.1. Based on an expected frequency of *T. gondii* exposure of 6.1% [48], a population size of 1,238,564 people aged 15 years and older in Durango, confidence limits of 1%, and a confidence level of 95%, the total sample size was calculated at 2197 individuals. The Pearson’s chi-square test was used to observe the association between two categorial variables. Logistic regression analysis with adjustment by age, gender, and residence area was performed to determine the association between *T. gondii* seropositivity and sexual promiscuity. Odd ratios (ORs) and 95% confidence intervals (CIs) were calculated, and statistical significance was set at *p* value < 0.05.

## 5. Conclusions

The results of this survey indicate that *T. gondii* seropositivity and serointensity are associated with sexual promiscuity. Further research on the association between *T. gondii* exposure and sexual promiscuity should be conducted.

## Figures and Tables

**Table 1:** A stratification by sex and age groups and the association between *T. gondii* seropositivity and sexual promiscuity.

	Sexual Promiscuity			No Sexual Promiscuity					
		Seropositivity			Seropositivity			95%	
	No.	To *T. gondii*		No.	To *T. gondii*			Confidence	*p*
Characteristic	Tested	No.	%	Tested	No.	%	OR	Interval	Value
Sex									
Male	94	30	31.9	748	104	13.9	2.90	1.79–4.69	<0.0001
Female	221	27	12.2	2870	270	9.4	1.34	0.87–2.04	0.17
Age (years)									
≤30	58	12	20.7	852	78	9.2	2.58	1.31–5.09	0.004
31–50	179	30	16.8	1785	197	11.0	1.62	1.06–2.45	0.02
>50	78	15	19.2	981	99	10.1	2.12	1.16–3.11	0.01
All	315	57	18.1	3618	374	10.3	1.91	1.41–2.60	<0.0001

**Table 2:** Stratification by sex and age groups and the association between high (>150 IU/mL) levels of anti-*T. gondii* IgG antibodies and sexual promiscuity.

	Sexual Promiscuity			No Sexual Promiscuity					
		>150 IU/mL			>150 IU/mL			95%	
	No.	of IgG		No.	of IgG			Confidence	*p*
Characteristic	Tested	No.	%	Tested	No.	%	OR	Interval	Value
Sex									
Male	94	15	16.0	748	40	5.3	3.36	1.77–6.35	<0.0001
Female	221	14	6.3	2870	127	4.4	1.46	0.82–2.58	0.19
Age (years)									
≤30	58	7	12.1	852	38	4.5	2.94	1.25–6.90	0.02
31–50	179	14	7.8	1785	86	4.8	1.67	0.93–3.01	0.08
>50	78	8	10.3	981	43	4.4	2.49	1.12–5.50	0.04
All	315	29	9.2	3618	167	4.6	2.09	1.38–3.16	0.0003

## Data Availability

Data are provided within the article.
